# Supplementary material for: Determinants of first-line antiretroviral treatment failure among adult patients on treatment in Mettu Karl Specialized Hospital, South West Ethiopia; a case control study
Source: PLoS One. 2021 Oct 22;16(10):e0258930. doi: 10.1371/journal.pone.0258930 (PMC8535443; doi:10.1371/journal.pone.0258930)
Supplement: Attachment — Submitted filename: PONE-D-21-13362 track changes.pdf [file pone.0258930.s003.pdf]

PLOS ONE

Determinants of first-line antiretroviral treatment failure among adult patients on antiretroviral therapy in a Specialized Hospital, South West Ethiopia; A case control Study

--Manuscript Draft--

Manuscript Number:	PONE-D-21-13362
Article Type:	Research Article
Full Title:	Determinants of first-line antiretroviral treatment failure among adult patients on antiretroviral therapy in a Specialized Hospital, South West Ethiopia; A case control Study
Short Title:	Determinants of first-line antiretroviral treatment failure in a Specialized Hospital, South West Ethiopia
Corresponding Author:	Sabit Zenu Siraj, Master of public health<br>Mettu University<br>Mettu, Oromia ETHIOPIA
Keywords:	HIV, First-line ART, Treatment failure, Mettu
Abstract:	Background: Antiretroviral therapy has dramatically reduced Human Immunodeficiency Virus related morbidity and mortality; and transformed HIV infection into a manageable chronic condition. However, first-line antiretroviral treatment failure continues to grow especially in resource limited settings. Despite this, determinants of first-line antiretroviral treatment failure are not well studied in Ethiopia.<br>Objective: To identify determinants of first-line antiretroviral treatment failure among adult patients on antiretroviral therapy in Mettu Karl Specialized Hospital, South West Ethiopia, 2020.<br>Methods: A hospital- based case-control study was conducted from October to November 2020. Simple random sampling technique was used to select participants. Interviewer administered questionnaire and record review were used for data collection. Data were entered into Epi data version 3.1 then exported to SPSS version 20 for analysis. Bivariable and multivariable logistic regression analysis were used. At the end, variables with P value < 0.05 at 95% confidence intervals for adjusted odds ratio were considered statistically significant.<br>Result: A total of 113 cases and 339 controls were included in the study with response rate of 98.6%. Sixty-four (56.6%) of cases and 183 (54.0%) of controls were females. Baseline WHO clinical stage III and IV (AOR =1.909, 95% CI: (1.103, 3.305), baseline body mass index<18.5kg/m 2 (AOR=2.208,95% CI: (1.257, 3.877),baseline CD4 cell count <100cells/mm 3 (AOR=3.016, 95% CI: (1.734, 5.246), having history of TB co-infection (AOR=1.855, 95% CI: (1.027, 3.353), having history of lost to follow up (AOR=3.235, 95% CI: (1.096, 9.551), poor adherence to medication (AOR=7.597, 95% CI: (4.059, 14.219), initiation of treatment after 2 years of diagnosis with HIV (AOR=4.979, 95% CI: (2.039, 12.158) were independently associated with first-line antiretroviral treatment failure.<br>Conclusion: In this study several variables were independently associated with first-line antiretroviral treatment failure. Concerned bodies should give more attention to early diagnosis of HIV, early enrollment in chronic HIV care and early initiation of ART before patients develop advanced WHO clinical stages. In addition, focus has to be given for patients with low CD4 count. Regular screening for TB, counseling on optimal adherence to medication and enhancing nutritional status of patients with low body mass index are crucial to prevent first-line antiretroviral treatment failure.
Order of Authors:	Sabit Zenu Siraj, Master of public health
	Tariku Tesema
	Mohammed Reshad Kedir
	Endegena Abebe
Additional Information:	
Question	Response

Powered by Editorial Manager® and ProduXion Manager® from Aries Systems Corporation

**Financial Disclosure**<br><br>Enter a financial disclosure statement that describes the sources of funding for the work included in this submission. Review the submission guidelines for detailed requirements. View published research articles from *PLOS ONE* for specific examples.<br><br>This statement is required for submission and **will appear in the published article** if the submission is accepted. Please make sure it is accurate.<br><br>**Unfunded studies**<br>Enter: *The author(s) received no specific funding for this work.*<br><br>**Funded studies**<br>Enter a statement with the following details:<br>• Initials of the authors who received each award<br>• Grant numbers awarded to each author<br>• The full name of each funder<br>• URL of each funder website<br>• Did the sponsors or funders play any role in the study design, data collection and analysis, decision to publish, or preparation of the manuscript?<br>• **NO** - Include this sentence at the end of your statement: *The funders had no role in study design, data collection and analysis, decision to publish, or preparation of the manuscript.*<br>• **YES** - Specify the role(s) played.<br><br>* typeset	The funders had no role in study design, data collection and analysis, decision to publish, or preparation of the manuscript.
**Competing Interests**<br><br>Use the instructions below to enter a competing interest statement for this submission. On behalf of all authors, disclose any competing interests that could be perceived to bias this work—acknowledging all financial support and any other relevant financial or non-financial competing interests.<br><br>This statement is required for submission	The authors have declared that no competing interests exist.

and **will appear in the published article** if the submission is accepted. Please make sure it is accurate and that any funding sources listed in your Funding Information later in the submission form are also declared in your Financial Disclosure statement.<br><br>View published research articles from *PLOS ONE* for specific examples.<br><br>**NO authors have competing interests**<br><br>Enter: *The authors have declared that no competing interests exist.*<br><br>**Authors with competing interests**<br><br>Enter competing interest details beginning with this statement:<br><br>*I have read the journal's policy and the authors of this manuscript have the following competing interests: [insert competing interests here]*<br><br>* typeset	
**Ethics Statement**<br><br>Enter an ethics statement for this submission. This statement is required if the study involved:<br><br>• Human participants<br>• Human specimens or tissue<br>• Vertebrate animals or cephalopods<br>• Vertebrate embryos or tissues<br>• Field research<br><br>Write "N/A" if the submission does not require an ethics statement.<br><br>General guidance is provided below. Consult the submission guidelines for detailed instructions. **Make sure that all information entered here is included in the Methods section of the manuscript.**	The study has been approved by ethics review comittee of College of Health Sciences, Mettu University. Individual participants provided informed consent.

**Format for specific study types**<br><br>**Human Subject Research (involving human participants and/or tissue)**<br>• Give the name of the institutional review board or ethics committee that approved the study<br>• Include the approval number and/or a statement indicating approval of this research<br>• Indicate the form of consent obtained (written/oral) or the reason that consent was not obtained (e.g. the data were analyzed anonymously)<br><br>**Animal Research (involving vertebrate animals, embryos or tissues)**<br>• Provide the name of the Institutional Animal Care and Use Committee (IACUC) or other relevant ethics board that reviewed the study protocol, and indicate whether they approved this research or granted a formal waiver of ethical approval<br>• Include an approval number if one was obtained<br>• If the study involved *non-human primates*, add *additional details* about animal welfare and steps taken to ameliorate suffering<br>• If anesthesia, euthanasia, or any kind of animal sacrifice is part of the study, include briefly which substances and/or methods were applied<br><br>**Field Research**<br><br>Include the following details if this study involves the collection of plant, animal, or other materials from a natural setting:<br>• Field permit number<br>• Name of the institution or relevant body that granted permission	
**Data Availability**<br><br>Authors are required to make all data underlying the findings described fully available, without restriction, and from the time of publication. PLOS allows rare exceptions to address legal and ethical concerns. See the PLOS Data Policy and FAQ for detailed information.	Yes - all data are fully available without restriction

A Data Availability Statement describing where the data can be found is required at submission. Your answers to this question constitute the Data Availability Statement and **will be published in the article**, if accepted.<br><br>**Important:** Stating 'data available on request from the author' is not sufficient. If your data are only available upon request, select 'No' for the first question and explain your exceptional situation in the text box.<br><br>Do the authors confirm that all data underlying the findings described in their manuscript are fully available without restriction?	
**Describe where the data may be found in full sentences. If you are copying our sample text, replace any instances of XXX with the appropriate details.**<br><br>• If the data are **held or will be held in a public repository**, include URLs, accession numbers or DOIs. If this information will only be available after acceptance, indicate this by ticking the box below. For example: *All XXX files are available from the XXX database (accession number(s) XXX, XXX.*).<br>• If the data are all contained **within the manuscript and/or Supporting Information files**, enter the following: *All relevant data are within the manuscript and its Supporting Information files.*<br>• If neither of these applies but you are able to provide **details of access elsewhere**, with or without limitations, please do so. For example:<br><br>*Data cannot be shared publicly because of [XXX]. Data are available from the XXX Institutional Data Access / Ethics Committee (contact via XXX) for researchers who meet the criteria for access to confidential data.*<br><br>*The data underlying the results presented in the study are available from (include the name of the third party*	All data is available from the corresponding author

and contact information or URL).<br>• This text is appropriate if the data are owned by a third party and authors do not have permission to share the data.<br><br>* typeset	
Additional data availability information:	

Manuscript

Click here to access/download;Manuscript;Final ART failure for PLOS.docx

Determinants of first-line antiretroviral treatment failure among adult patients on antiretroviral therapy in a Specialized Hospital, South West Ethiopia; A case control Study

Authors

Sabit Zenu Siraj[¶1*] Tariku Tesema[¶2], Mohammed Reshad Kedir[¶3], Endegena Abebe[¶4]

1. MPH in Epidemiology, Lecturer and Researcher in the department of Public Health, College of Health Sciences, Mettu University

   *Corresponding Author
2. MPH, Instructor at Mettu College of Health Sciences, Mettu
3. MPH in Epidemiology, Lecturer and Researcher in the department of Public Health, College of Health Sciences, Mettu University
4. PhD, Assistant Professor of Physiology in the Department of Bio Medical Sciences, College of Health Sciences, Mettu University

*Corresponding Author

Email: sabitzeinu91@gmail.com (SZ)

¶ These Authors made equal collaborations

Abstract

Background: Antiretroviral therapy has dramatically reduced Human Immunodeficiency Virus related morbidity and mortality and transformed HIV infection into a manageable chronic condition. However, first-line antiretroviral treatment failure continues to grow especially in resource limited settings. Despite this, determinants of first-line antiretroviral treatment failure are not well studied in Ethiopia.

Objective: To identify determinants of first-line antiretroviral treatment failure among adult patients on antiretroviral therapy in Mettu Karl Specialized Hospital, South West Ethiopia, 2020.

Methods: A Hospital- based case-control study was conducted from October to November 2020. Simple random sampling technique was used to select participants. Interviewer administered questionnaire and record review were used for data collection. Data were entered into Epi data version 3.1 then exported to SPSS version 20 for analysis. Bivariable and multivariable logistic regression analysis were used. At the end, variables with P value < 0.05 at 95% confidence intervals for adjusted odds ratio were considered statistically significant.

Result: A total of 113 cases and 339 controls were included in the study with response rate of 98.6%. Sixty-four (56.6%) of cases and 183 (54.0%) of controls were females. Baseline WHO clinical stage III and IV (AOR =1.909, 95% CI: (1.103, 3.305), baseline body mass index<18.5kg/m^2(AOR=2.208,95% CI: (1.257, 3.877),baseline CD4 cell count <100cells/mm^3(AOR=3.016, 95% CI: (1.734, 5.246), having history of TB co-infection (AOR=1.855, 95% CI: (1.027, 3.353), having history of lost to follow up (AOR=3.235, 95% CI: (1.096, 9.551), poor adherence to medication (AOR=7.597, 95% CI: (4.059, 14.219), initiation of treatment after 2 years of diagnosis with HIV (AOR=4.979, 95% CI: (2.039, 12.158) were independently associated with first-line antiretroviral treatment failure.

Conclusion: In this study several variables were independently associated with first-line antiretroviral treatment failure. Concerned bodies should give more attention to early diagnosis of HIV, early enrollment in chronic HIV care and early initiation of ART before patients develop advanced WHO clinical stages. In addition, focus has to be given for patients with low CD4 count. Regular screening for TB, counseling on optimal adherence to medication and enhancing nutritional status of patients with low body mass index are crucial to prevent first-line antiretroviral treatment failure.

Key words: HIV, First-line ART, Treatment failure, Mettu

Introduction

Human Immunodeficiency Virus (HIV) epidemic has become one of the world's public health problems and development challenges (1). Approximately 76 million people have been infected with HIV since the start of HIV pandemic. In 2019, there were 38 million people living with HIV, 1.7 million new infections and 690,000 deaths due to Acquired immune deficiency syndrome (AIDS) (2).

Antiretroviral therapy has dramatically reduced HIV related morbidity and mortality and has transformed HIV infection into a manageable chronic condition; it is also highly effective at reducing sexual transmission of HIV in patients who have adequately suppressed viral loads (3). The ART treatment goals are suppression of HIV replication, restoration and preservation of immune function, reduction in HIV-related morbidity and mortality that improves the quality of life people living with HIV/AIDS (PLWHA) (4). On the other hand, when first-line antiretroviral treatment failure develops, all benefits of ART are affected (5).

Virological suppression, clinical and immunological improvement are expected from PLWHA after initiation of ART (3). First-line ART failure occurs when a combination of the antiretroviral regimen fails to control HIV infection. This could be virologic, immunologic and/or clinical failure (6). Virologic failure occurs when plasma viral load remains above 1000 copies/ml based on two consecutive viral load measurements in three-month interval, with adherence support following the first-viral load test. Immunological failure occurs when CD4 count at or below 250 cells/mm^3 following clinical failure or persistent CD4 levels below 100cells/mm^3. Clinical failure occurs when new or recurrent clinical event indicating severe immunodeficiency (WHO clinical stage 4 condition) occurs after 6 months of effective treatment (4).

Frequent assessment of treatment response is important while the subject is on ART. Monitoring the response to ART and diagnosis of treatment failure for patients on antiretroviral therapy is important to achieve treatment goals of ART. First-line antiretroviral treatment failure can be assessed virologically, immunologically and or clinically (5).

WHO recommended viral load testing as the preferred monitoring approach to diagnose and confirm antiretroviral treatment failure in 2013 (7). However, the availability of viral load testing is very limited in low- and middle-income countries, where the majority of people

living with HIV reside (8). Viral load provides an early and more accurate indication of treatment failure when compared with clinical and immunological monitoring (4).

Globally, about 10-20% of adult patients on first-line antiretroviral treatment are reported to have developed treatment failure with higher figures (15-25%) being reported in Sub-Saharan Africa (9). In Sub-Saharan Africa, many patients who experience treatment failure do not switch to potent second-line regimens due to resource limitation, yet those who remain on failing first-line regimen experience disproportionately higher morbidity and mortality compared to those who switch (10). In Ethiopia, prevalence of first-line antiretroviral treatment failure was 15.3% by using the three WHO treatment failure criteria (virological, immunological and clinical)(11).

Despite the scaling up of antiretroviral treatment in resource limited settings, development of first-line antiretroviral treatment failure is a big challenge(12). Treatment failure among population taking ART in Ethiopia is a public health concern because patients experiencing treatment failure will have increased risk of morbidity, mortality and increased transmission as well as accumulation of drug resistant mutations (13).

According to a study conducted in the United States(US) in 2014, the cost of treating a patient with a second-line ART drug increases by 24% as compared with the first-line treatment(14). Currently, in Ethiopia where medication is fully funded by the government, treatment failure and frequent substitution of medications are becoming major challenges in control of the disease (15).

Different studies identified that age <35 years, being male, high educational level, urban residence, unemployment, advanced clinical stage III/IV, having history of TB co-infection, baseline CD4< 100cell/mm3, baseline BMI <18.5 kg/mm2, poor adherence, lost to follow up, baseline ART regimen, high frequency of alcohol use and smoking as determinant factors of first-line antiretroviral treatment failure (16–20).

Investigating and managing determinant factors of first-line antiretroviral treatment failure is very important to achieve treatment targets, decrease morbidity and mortality, decrease HIV transmission and sustain the quality of life of PLWHA.

The need of undertaking the study is that there is limited evidence on determinants of first-line antiretroviral treatment failure in Ethiopia and no known research has been done in the study area to identify determinant factors of first-line antiretroviral treatment failure among

adult patients on ART. Even though viral load test is the gold standard technique for early detection of first-line antiretroviral treatment failure, most of the previous studies in Ethiopia, did not consider virological failure because of the absence of the test service in primary care settings. Many studies consider only immunological and clinical failure as criteria of first-line antiretroviral treatment failure. But in this study virological failure, which is one of the decision criteria of first-line antiretroviral treatment failure was considered in addition to immunological and clinical failure. Therefore, this study aimed to identify determinants of first-line antiretroviral treatment failure among adult patients on antiretroviral therapy in Mettu Karl Referral Hospital, South West Ethiopia.

Materials and Methods

Study design, area and period

A Hospital based unmatched case-control study was conducted. The study was conducted at Mettu Karl Specialized Hospital which the only specialized in the area serving a population of more than 1.4 million people with different services including HIV prevention, care and treatment. In this hospital ART service started in 2005. Currently total people receiving ART at the facility is 1600. Mettu Karl Specialized Hospital has laboratory services to determine CD4 count and viral load to monitor ART patients. The study was conducted from October 25 to November 24/2020.

Source and Study populations

Source populations

The source populations for cases in the study were all adult PLWHA documented to have first-line antiretroviral treatment failure and enrolled to the second-line antiretroviral treatment at the hospital. The source populations for controls of this study were all adult PLWHA who did not develop first-line antiretroviral treatment failure and on first-line antiretroviral treatment the hospital

Study populations

Study populations for cases in the study were adult HIV Patients on ART, aged $\geq$ 18 years, documented to have first-line antiretroviral treatment failure and eligible for the study during study period. Study population for controls were adult HIV patients on ART, aged $\geq$18 years, who did not fail first-line antiretroviral treatment and who were on first-line ART for six or more months

Sample size determination and sampling technique

Sample size was calculated using Epi Info version 7 for unmatched case control study design by considering the significant determinant factor of first-line antiretroviral treatment failure such as baseline BMI< 18.5kg/m^2, having history of lost to follow up, baseline CD4 cell<50 cell/mm^3, poor adherence, smoking, history of TB co-infection.

Since, poor adherence was found to result in the largest sample size; it was used to determine sample size as independent variable. In previously conducted research proportion of poor adherence was 22.9% in cases and 10.8% in controls (19). By using 5 % margin of error, 80% power, a case to control ratio of 1:3 and using a two population proportion formula, the

calculated sample size was 416 (104 cases and 312 controls). Then by adding a 10% non-response rate, the final sample size was 458 (115 cases and 343 controls).

Simple random sampling technique was used to recruit study subjects for cases and controls. Adult patients who developed first-line antiretroviral treatment failure and those adult patients did not develop first-line antiretroviral treatment failure were identified and their medical record number (MRN) were listed as cases and controls. Accordingly, register of 1353 patients who did not develop first-line antiretroviral treatment failure and on first-line was developed. Then to select study subjects for controls simple random sampling technique was used by computer generated random numbers based on medical record number (MRN) of patients.

For cases, register of 194 adult patients who developed first-line antiretroviral treatment failure and on second-line antiretroviral therapy at the hospital was prepared. Cases were then selected by simple random sampling technique using computer generated random numbers based on medical record number (MRN) of patients.

Data collection tools and procedures

Data was collected from patients and medical records using structured interviewer administered questionnaire and structured checklists respectively. The questionnaire was developed from WHO ART guideline, ART follow up guideline of Federal Ministry of Health of Ethiopia and EDHS 2016 (4,5,21). In addition, other tools were adopted from previously conducted studies (22–25). The questionnaire comprised four parts; socio-demographic factors, clinical factors, antiretroviral treatment related factors and behavioral factors. Questions related with behavioral factors and socio-demographic factors were included in structured interviewer administered questionnaires and collected from patients. Questions related to clinical and antiretroviral treatment related factors were included in the checklist and collected from record review.

Data analysis procedures

The collected data was coded and entered into Epi data version 3.1 and exported to statistical package for social sciences (SPSS) version 20 for analysis. Checking and cleaning of data was done before analysis. Frequencies and proportions were used to describe the data. Cross tabulations was used to summarize descriptive statistics in each group. Bivariable and Multivariable logistic regression were used to identify determinants of first-line antiretroviral treatment failure. Variables with P-value <0.25 at bivariable analysis were taken to

multivariable logistic regression analysis. The multivariable model was fitted to identify the independent determinants of first-line antiretroviral treatment failure using backward stepwise removal method. The model fitness was checked by Hosmer-Lemeshow goodness of fit test by considering p-value >0.05. Finally, variables that have significant association with first-line antiretroviral treatment failure were identified and reported based on the adjusted odds ratio (AOR) with corresponding 95% CI at P-value <0.05. The final finding was presented by description and tables.

Result

Socio-demographic characteristics of participants

A total of 452(113 cases and 339 controls) HIV patients on ART were involved in the study with response rate of 98.6%. Sixty-four (56.6%) of cases and 183 (54.0%) of controls were females. Regarding marital status, 42 (37.2%) of cases and 193 (56.9%) of controls were married; while 30 (26.5%) of cases and 33 (9.7%) of controls were single. Forty-one (36.3%) of cases and 92 (27.1%) of controls were living in rural areas (Table 1).

Table 1: Socio-demographic Characteristics of HIV Patients on ART at Mettu Karl Referral Hospital, South West Ethiopia, 2020

**Variable**	**Category**	**Cases (%) (n=113)**	**Control (%) (n=339)**	**Total (%)**
Age at initiation of ART	< 35 years	49 (43.4%)	119 (35.1%)	168 (37.2%)
	>=35 years	64(56.6%)	220 (64.9%)	284 (62.8%)
Sex	Male	49 (43.4%)	156 (46.0%)	205 (45.4%)
	Female	64 (56.6%)	183 (54.0%)	247 (54.6%)
Marital status	Married	42 (37.2%)	193 (56.9%)	235 (52%)
	Single	30 (26.5%)	33 (9.7%)	63 (13.9%)
	Divorced	25 (22.1%)	63 (18.6%)	88 (19.5%)
	Widowed	16 (14.2%)	50 (14.7%)	66 (14.6%)
Place of residence	Urban	72 (63.7%)	247 (72.9%)	319 (70.6%)
	Rural	41 (36.3%)	92 (27.1%)	133 (29.4%)
Occupational status	Government employee	13 (11.5%)	37 (10.9)	50 (11.1%)
	Farmer	20 (17.7%)	45 (13.3%)	65 (14.4%)
	Daily laborer	21 (18.6%)	57 (16.8%)	78 (17.3%)
	Merchant	21 (18.6%)	79 (23.3%)	100 (22.1%)
	House wife	22 (19.5%)	95 (28%)	117 (25.9%)
	Student	7 (6.2%)	9 (2.7%)	16 (3.5%)
	Others*	9 (8.0%)	17 (5.0%)	26 (5.8%)
Educational status	No formal education	21 (18.6%)	60 (17.7%)	81 (17.9%)
	Primary school (1-8)	41 (36.3%)	169 (49.9%)	210 (46.5%)
	Secondary school (9-12)	33 (29.2%)	67 (19.8%)	100 (22.1%)
	College and above	18 (15.9%)	43 (12.7%)	61 (13.5%)
Criteria used to diagnose treatment failure	Virological	24 (21.2%)		
	Clinical/immunological	39 (34.5%)		
	Clinical/virological	8 (7.1%)		
	Immunological/virological	7 (6.2%)		
	All criteria	35 (31.0%)		

*Driver, self and private business, NGO

Bivariable logistic regression analysis of first-line antiretroviral treatment failure

Bivariable logistic regression analysis was carried out to assess the association of variables with first-line treatment failure. Among these variables, smoking, khat chewing, baseline WHO clinical stage, baseline BMI, baseline CD4 count, history of TB co-infection, history of lost to follow up, adherence status to antiretroviral drugs, disclosure status, time lag to initiate ART after diagnosis with HIV were candidates for multivariable logistic regression analysis at P-value <0.25 in bivariable logistic regression model (Table 2).

Table 2 Bivariate Logistic Regression Analysis of First-line Antiretroviral Treatment Failure of HIV Patients on ART at Mettu Karl Referral Hospital, South West Ethiopia, 2020

**Variable**	**Category**	**Case (N=113)**	**Control (N=339)**	**COR (95% CI)**	**P-value**
Smoking status	Yes	28 (24.8%)	57 (16.8%)	1.630 (0.975, 2.723)	.062
	No	85 (75.2%)	282 (83.2%)	**1**	
Khat chewing	Yes	30 (26.5%)	67 (19.8%)	1.462 (0.890, 2.400)	.133
	No	83 (73.5%)	271 (80.2%)	**1**	
Baseline WHO stage	Stage I and II	53 (46.9%)	234 (69.0%)	**1**	
	Stage III and IV	60 (53.1%)	105 (31.0%)	2.523 (1.632, 3.899)	.000
Baseline BMI	<18.5kg/m^2	63 (55.8%)	89 (26.3%)	3.539 (2.272, 5.513)	.000
	>=18.5kg/m^2	250 (73.7%)	50 (44.2%)	**1**	
Baseline CD4 cell count	<100cells/mm^3	59(52.2%)	69 (20.4%)	4.275 (2.715, 6.732)	.000
	>=100cells/mm^3	54 (47.8%)	270 (79.6%)	**1**	
TB co-infection	Yes	46 (40.7%)	65 (19.2%)	2.894 (1.822, 4.596)	.000
	No	67 (59.3%)	274 (80.8%)	**1**	
History of lost to follow up	Yes	15 (13.3%)	8 (2.4%)	6.333 (2.608, 15.378)	.000
	No	98 (86.7%)	331 (97.6%)	**1**	
Adherence status	Good	37 (32.7%)	240 (70.8%)	**1**	
	Fair	18 (15.9%)	65 (19.2%)	1.796 (0.960, 3.360)	.067
	Poor	58 (51.3%)	34 (10.0%)	11.065 (6.404, 19.118)	.000
Disclosure status	Yes	105(92.9%)	334 (98.5%)	**1**	
	No	8 (7.1%)	5 (1.5%)	5.090 (1.630, 15.893)	.005
Time lag to initiate ART after diagnosis with HIV	Within the same month	30 (26.5%)	158 (46.6%)	**1**	
	One to twenty four months	65 (57.5%)	157 (46.3%)	2.180 (1.342, 3.544)	.002
	After twenty four months	18 (15.9%)	24 (7.1%)	3.950 (1.913, 8.157)	.000

COR: crude odds ratio, CI: confidence interval, 1: reference category

Determinants of first-line antiretroviral treatment failure

In multivariable logistic regression analysis, baseline WHO clinical stage III and IV, baseline body mass index < 18.5kg/m^2, baseline CD4 count <100 cells/mm^3, having history of TB co-infection, having history of lost to follow up, poor adherence to antiretroviral drugs, initiation of ART after two years of diagnosis with HIV were significantly associated with first-line antiretroviral treatment failure.

The finding of this study showed that, HIV positive patients on ART with stages III and IV baseline WHO clinical stage were almost two times more likely to fail first-line antiretroviral treatment when compared with stage I and II baseline WHO clinical stage [AOR =1.909, 95% CI: 1.103, 3.305].

Patients on ART with baseline Body Mass Index of <18.5kg/m^2 were two times more likely to develop first-line antiretroviral treatment failure than patients with baseline BMI of >=18.5kg/m^2 [AOR=2.208, 95% CI: 1.257, 3.877].

Patients on ART withCD4 count of <100cells/mm^3 at the time of initiation of ART had three times more likelihood to fail first-line antiretroviral treatment when compared with those who had CD4 count of >=100cells/mm^3 at the time of initiation of ART [AOR=3.016, 95% CI: 1.734, 5.246].

Patients with history of TB co-infection were almost twice more likely to develop first-line treatment failure than those patients without TB co-infection [AOR=1.855, 95% CI: 1.027, 3.353].

Patients with history of lost to follow up had three times more likely to fail first-line antiretroviral treatment when compared with patients did not have lost to follow up history [AOR=3.235, 95% CI: 1.096, 9.551].

HIV positive patients on ART with poor adherence to antiretroviral drugs were more than seven times more likely to fail first-line antiretroviral treatment when compared to patients with good adherence to antiretroviral drugs [AOR=7.597, 95% CI: 4.059, 14.219].

Patients who initiated ART after two years of being HIV positive had around five times more probability to fail first-line antiretroviral treatment when compared to patients initiated ART within the same month of being HIV positive [AOR=4.979, 95% CI: 2.039, 12.158] (Table 3).

Table 3 Multivariable Logistic Regression Analysis on Determinants of First-line Antiretroviral Treatment Failure among HIV Patients on ART at Mettu Karl Referral Hospital, South West Ethiopia, 2020

Variable	Category	Case (N=113)	Control (N=339)	COR (95% CI)	AOR (95% CI)
Smoking status	Yes	28 (24.8%)	57 (16.8%)	1.630 (0.975, 2.723)	1.231 (0.542, 2.792
	No	85 (75.2%)	282 (83.2%)	1	1
Khat chewing	Yes	30 (26.5%)	67 (19.8%)	1.462 (0.890, 2.400)	1.243 (0.570, 2.711)
	No	83 (73.5%)	271 (80.2%)	1	1
Baseline WHO clinical stage	Stage I and II	53 (46.9%)	234 (69.0%)	1	1
	Stage III and IV	60 (53.1%)	105 (31.0%)	2.523 (1.632, 3.899)	**1.909 (1.103, 3.305)***
Baseline BMI	<18.5kg/m^2	63 (55.8%)	89 (26.3%)	3.539 (2.272, 5.513)	**2.208 (1.257, 3.877)***
	>=18.5kg/m^2	250 (73.7%)	50 (44.2%)	1	1
Baseline CD4	<100 Cells/mm3	59(52.2%)	69 (20.4%)	4.275 (2.715, 6.732)	**3.016 (1.734, 5.246)***
	>=100Cells/mm^3	54 (47.8%)	270 (79.6%)	1	1
TB co-infection	Yes	46 (40.7%)	65 (19.2%)	2.894 (1.822, 4.596)	**1.855 (1.027, 3.353)***
	No	67 (59.3%)	274 (80.8%)	1	1
History of lost to follow up	Yes	15 (13.3%)	8 (2.4%)	6.333(2.608, 15.378)	**3.235(1.096, 9.551)***
	No	98 (86.7%)	331 (97.6%)	1	1
Adherence status	Good	37 (32.7%)	240 (70.8%)	1	1
	Fair	18 (15.9%)	65 (19.2%)	1.796 (0.960, 3.360)	1.322 (0.643, 2.716)
	Poor	58 (51.3%)	34 (10.0%)	11.065(6.404, 19.118)	**7.597 (4.059, 14.219)***
Disclosure status	Yes	105 (92.9%)	334 (98.5%)	1	1
	No	8 (7.1%)	5 (1.5%)	5.090 (1.630, 15.893)	1.663 (0.460, 6.013)
Time lag to initiate ART after diagnosis with HIV	Within the same month	30 (26.5%)	158 (46.6%)	1	1
	One to twenty four months	65 (57.5%)	157 (46.3%)	2.180 (1.342, 3.544)	1.702 (0.928, 3.121)
	After twenty four months	18 (15.9%)	24 (7.1%)	3.950 (1.913, 8.157)	**4.979 (2.039, 12.158)***

**Statistically significant at p-value<0.05, COR: crude odds ratio, AOR: adjusted odds ratio, CI: confidence interval, 1: reference category*

Discussion

The identification and management of first-line ART failure is a key challenge for HIV programs in resource-limited settings. Staying on a failing first-line antiretroviral therapy is associated with an increased risk of mortality. In addition to this, development of drug resistance limits the ability to construct new, potent, and tolerable regimens in the future. This study was aimed to identify determinants of first-line antiretroviral treatment failure.

In this study first-line antiretroviral treatment failure was found to be significantly associated with stage III and IV baseline WHO clinical stage of HIV, low baseline Body Mass Index (<18.5 kg/m^2), baseline CD4 count <100cells/mm^3, having history of TB co-infection, having history of lost to follow up, poor adherence to antiretroviral drugs and initiation of ART after two years of diagnosis with HIV positive.

The current study has identified that patients with advanced WHO clinical stage III and IV at the time of initiation of ART were almost two times more likely to fail first-line antiretroviral treatment when compared with stage I and II baseline WHO clinical stage. This finding has similarity with the studies conducted in Sanglah Hospital, Bali Indonesia(17),Senegal(26), and in Harar public hospitals, Eastern Ethiopia(23) . This might be due the fact that advanced WHO clinical stage of HIV disease is associated with high viral load and low CD4 cell count that compromise immunity and may negatively affect response to first-line antiretroviral treatment (27).

In this study patients with baseline low Body Mass Index of <18.5kg/m^2 were two times more likely to develop first-line antiretroviral treatment failure than patients with baseline Body Mass Index of >=18.5kg/m^2. This finding is consistent with studies done in Woldia Hospital, North East Ethiopia, by 2017 (22), University of Gonder Specialized Hospital, Ethiopia, in 2019 (28). This might be due to the fact that patients with low body mass index (BMI<18.5kg/m2) have low nutritional status that leads to weakened immunity, blunted immune response and increased risk of first-line antiretroviral treatment failure (29).

The finding of this study indicated that patients with low baseline CD4 count <100cells/mm^3 were three times more likely to fail first-line antiretroviral treatment than patients with baseline CD4 count >=100cells/mm^3. This finding is comparable with studies done in India by 2016 (30), in Northwestern Tanzania by 2019 (12), in Dire Dawa, Eastern Ethiopia by 2019 (31). This finding might be due the reason that patient with low baseline CD4 cell count have a lesser immunity that may favor the occurrence of opportunistic infection and lead to

clinical failure. And also, this low baseline CD4 cell count is difficult to be replaced enough in HIV patients on ART and may lead to first-line antiretroviral treatment failure (27,32)

Having history of TB co-infection was independently associated with first-line antiretroviral treatment failure. Patients who had history of TB co-infection were around two times more likely to fail first-line antiretroviral treatment when compared to patients who had no history of TB co- infection. Similarly studies conducted in India by 2016 (30), Gutu District, Zimbabwe by 2017 (16), Debre Markos Referral Hospital, North West Ethiopia by 2018 (33), showed that HIV-TB co-infection was independent determinant factors of treatment failure among adult patients on ART. The occurrence of tuberculosis during antiretroviral treatment has multiple effects including pills burden and drug-drug interaction which may lead to first-line treatment failure(34).

Having history of lost to follow up was associated with first-line treatment failure. Patients with history of lost to follow up were three times more likely to develop first-line treatment failure than patients without history of lost to follow up. This finding is similar with studies conducted in Harare Central Hospital, Zimbabwe by 2014 (9), in Nigerian Teaching Hospital, Nigeria by 2019 (35), in Central Ethiopia St. Luke Referral Hospital and Tulu bolo General Hospital by 2019 (36). This might be due to the result of on-going viral replication in the absence of ART, which results in decrease of CD4 cell count and increase viral load that leads to first-line antiretroviral treatment failure (37).

Patients on ART with poor adherence to antiretroviral drugs have more than seven times higher probability of developing first-line treatment failure than patients with good adherence to antiretroviral drugs. The finding is consistent with studies conducted in Nigerian Teaching Hospitals by 2019 (35), in Cameroon by 2016 (38), University of Gondar Teaching Hospital by 2016 (24), in Sekota, Northeast Ethiopia by 2019 (39). This might be due to the fact that adherence issue is the pillar for patients on ART. Patients with poor adherence to antiretroviral drugs are associated with loss of sustained viral suppression, higher risk of drug resistance that leads to first-line antiretroviral treatment failure(40).

Patients who were enrolled to ART after two years of diagnosis with HIV were nearly five times more likely to develop first-line antiretroviral treatment failure when compared with patients who were enrolled to ART within the same month of being diagnosed with HIV. This finding was in line with those studies conducted in Zimbabwe (9), in Central Ethiopia St. Luke Referral Hospital and Tulu bolo General Hospital (36). This might be due to the

possibility that patients who stay long time without initiation of ART after diagnosis face an increase in viral load and develop other opportunistic infections. This might also be due to the difficulty of viral suppression and increase CD4 cell count when the patients delay to start ART (41).

Conclusion

This study showed that baseline stage III and IV WHO clinical stage of HIV, low baseline Body Mass Index (<18.5 kg/m^2), baseline CD4 count <100cells/mm^3, having history of TB co-infection, having history of lost to follow up, poor adherence to antiretroviral drugs and initiation of ART after two years of diagnosis with HIV positive were factors associated with first-line antiretroviral treatment failure.

Acknowledgement

We would like to thank Mettu University College of Health Sciences, Department of Public Health for giving us all necessary cooperation to undertake this study. Our gratitude also goes to data collectors, supervisors and study participants for their commitment and cooperation.

We also express our sincere thanks to our friends for their direct and indirect contribution in finalization of this research work

References

1. UNAIDS. Communities at the Centre of defending rights, breaching barriers and reaching people with HIV services. Glob AIDS Updat 2019.

2. UNAIDS. Seizing The Moment: Tackling entrenched inequalities to end epidemic. Global AIDS Update. 2020.

3. DHHS– (OARAC). Guidelines for the Use of Antiretroviral Agents in Adults and Adolescents with HIV. AIDS info. 2019. 13–35 p.

4. WHO. Consolidated guidelines on the use of antiretroviral drugs for treating and preventing HIV infection. 2nd ed. Geneva, Switzerland: WHO; 2016. 71–150 p.

5. Federal Ministry of Health-Ethiopia. National Consolidated Guidelines for Comprehensive HIV Prevention, Care and Treatment. 2nd ed. Addis Ababa: FMOH-Ethiopia; 2018. 42–82 p.

6. U.S. Department of Health and Human Services. Glossary of HIV/AIDS-Related Terms. 9th ed. U.S. Department of Health and Human Services, editor. Rockville: AIDSinfo; 2018. 180–183 p.

7. Dr. Jeremy Nel. HIV Resistance and Treatment Failure. Dep Infect Dis. 2015;(May).

8. UNAIDS. The need for routine viral load testing: Questions and Answers. UNAIDS; 2016. 1–12 p.

9. Chawana T, Reid A, Bwakura T, Gavi S, Nahachi S. Factors Influencing Treatment failure in HIV Positive Adult Patients on First Line Antiretroviral Therapy. Cent Afr J Med. 2014;

10. Kwobah C, Mwangi A, Koech J SG. Factors Associated with First-Line Antiretroviral Therapy Failure amongst HIV-Infected African Patients : A Case-Control Study. World J AIDS. 2012;2:271–8.

11. Assemie M, Alene M, Ketema D MS. Treatment failure and associated factors among first line patients on highly active antiretroviral therapy in Ethiopia : a systematic review and meta-analysis. Glob Heal Res Policy. 2019;4(32):1–10.

12. Gunda D, Kilonzo S, Kamugisha E, Meda J MB. Prevalence and Predictors of Virological Failure among Adults HIV Patients Receiving First Line ART in Northwestern Tanzania: A Cross Sectional Study. EC Microbiol. 2019;15(9):683–90.

13. Getaneh Y, Egziabiher A ZK et al. HIV-1 Treatment Failure among Population Taking Antiretroviral Therapy in Ethiopia. J AIDS HIV Treat. 2019;1(2):46–57.

14. Solem CT, Snedecor SJ, Khachatryan A, Nedrow K. Cost of Treatment in a US Commercially Insured , HIV-1 – Infected Population. PLoS One. 2014;9(5):e98152.

15. Tagar E, Sundaram M, Condliffe K, Matatiyo B, Chimbwandira F, Chilima B, et al. Multi-Country Analysis of Treatment Costs for HIV / AIDS (MATCH): Facility-Level ART Unit Cost Analysis in Ethiopia, Malawi, Rwanda, South Africa and Zambia. PLoS One. 2014;9(11).

16. Zikiti A, Maradzika JC, Marume A, Chapoterera B. Predictors of First Line Antiretroviral Therapy (ART) Failure among ART Patients in Gutu District, Masvingo Province, Zimbabwe. Int J Heal Sci Res. 2017;8(8):8–20.

17. Istri C, Dharma S, Sawitri AAS, M TP. Factors associated to first line antiretroviral therapy (ART) failure among HIV / AIDS patients at Sanglah Hospital , Bali. Public Heal Prev Med Arch. 2017;5(1):4–11.

18. Leng X, Liang S, Ma Y, Dong Y, Kan W, Xing H, et al. HIV virological failure and drug resistance among injecting drug users receiving fi rst-line ART in China. BMJ Open. 2014;4:e005886.

19. Meshesha HM, Nigussie ZM, Asrat A, Mulatu K. Determinants of virological failure among adults on first- line antiretroviral therapy at public health facilities in Kombolcha town , Northeast , Ethiopia : a case – control study. BMJ Open. 2020;10(e036223):1–9.

20. Aldous A. et al. Prevalence and trends in transmitted and acquired antiretroviral drug resistance ,. BMC Res Notes. 2017;1–10.

21. Ethiopian Central Statistical Agency. Ethiopian Demographic and Health survey. Addis Ababa: CSA; 2016. 120–146 p.

22. Babo Y, Alemie G, Fasil F. Predictors of first-line antiretroviral therapy failure amongst HIV-infected adult clients at Woldia Hospital , Northeast Ethiopia. PLoS One. 2017;12(11):1–19.

23. Feleke R, Geda B, Roba KT, Weldegebreal F. Magnitude of antiretroviral treatment failure and associated factors among adult HIV-positive patients in Harar public hospitals , Eastern Ethiopia. SAGE Open Med. 2018;8:1 –7.

24. Biset M, Kumilachew A, Samson G, Derso T, Desalegn E, Yemisirach T, et al. First-line antiretroviral treatment failure and associated factors in HIV patients at the University of Gondar Teaching Hospital , Gondar , Northwest Ethiopia. HIV/AIDS - Res Palliat Care. 2016;141–6.

25. Jaleta F, Getahun A, Bayissa, Getu. Determinants of First Line Anti-Retroviral Treatment Failure Among Adult Human Immunodeficiency Virus Infected Patients in Western Oromia Public Hospitals , West Ethiopia. Am J Heal Res. 2019;7(5):71–8.

26. Selly B, Ba ND, Sembene L, Dia H, Coulibaly M. Prevalence and Factors Associated with Virologic Failure among People Living with HIV (PLHIV) Monitored in a Decentralized Health Care Facility. Adv Infect Dis. 2019;9:226–37.

27. Palmisano L, Vella S. A brief history of antiretroviral therapy of HIV infection : success and challenges. aNN isT super saNiTà. 2011;47(1):44–8.

28. Agezew T, Tadesse A, Derseh L, Yimer M. Incidence and Predictors of First Line Anti-Retroviral Therapy Failure among Adults Receiving HIV Care in North West Ethiopia :A Hospiatal Based Follow up Study. Infect Dis Epidemiol. 2019;5(2):1–7.

29. Li X, Ding H, Geng W, Liu J, Jiang Y, Xu J. Predictive effects of body mass index on immune reconstitution among HIV-infected HAART users in China. BMC Infect Dis. 2019;19(373):1–9.

30. Rajian M, Gill P, Chaudhary, Uma. Prevalence of virological failure amongst WHO-defined immunological failure HIV patients on first line of highly active antiretroviral therapy in a tertiary care hospital in Haryana , India. Int J Res Med Sci. 2016;4(May):1613–6.

31. Lenjiso G, Endale B, Bacha Y, Dessie. Clinical and immunological failure among HIV-positive adults taking first-line antiretroviral therapy in Dire Dawa , eastern Ethiopia. BMC Public Health. 2019;1–10.

32. Gezie LD. Predictors of CD4 count over time among HIV patients initiated ART in Felege Hiwot Referral Hospital , northwest Ethiopia. BMC Res Notes. 2016;1–9.

33. Abebaw A. Magnitude of virologic failure and associated factors among adult patients on antiretroviral therapy at Debre Markos Referral Hospital , Northwest Ethiopia , 2018. Res Sq. 2018;1–34.

34. UNAIDS. Global AIDS Monitoring: Indicators for Monitoring the 2016 Political Declaration on Ending AIDS. UNAIDS; 2020.

35. Kwaghe V, Abdulrahman N, Kumtong K, Nkanga R. Prevalence and Risk Factors of First Line Antiretroviral Therapy Failure Amongst Adult HIV Patients at a Nigerian Teaching Hospital : A 10 Year Retrospective Study. Int Med Dent Res. 2019;6(4):13–7.

36. Mulisa D, Tesfa M, Kassa GM, Tolossa T. Determinants of first line antiretroviral therapy treatment failure among adult patients on ART at central Ethiopia : un-matched case control study. BMC Infect Dis. 2019;19(1024):1–13.

37. Opio D, Semitala FC, Kakeeto A, Sendaula E, Okimat P, Nakafeero B, et al. Loss to follow-up and associated factors among adult people living with HIV at public health facilities in Wakiso district , Uganda. BMC Heal Serv Res https//doi.org/101186/s12913-019-4474-6 19628 Res. 2019;6:1–10.

38. Patient J, Enoné M, Penda CI, Ebong SB, Mbangue M, Mandengué SH, et al. First-line antiretroviral treatment and virological response among HIV adult patients at an accredited HIV care center , Cameroon. Transl Med Commun. 2016;9:1–11.

39. Nega J, Taye S, Million Y, Rodrigo C, Eshetie S. Antiretroviral treatment failure and associated factors among HIV patients on first ‐ line antiretroviral treatment in Sekota , northeast Ethiopia. AIDS Res Ther. 2019;1–9.

40. Tadesse S, Tadesse A, Wubshet, Mamo. Adherence to Antiretroviral Treatment and Associated Factors among People Living with HIV / AIDS in Northwest Ethiopia. J Trop Dis. 2014;2(2).

41. World Health Organization (WHO). Guideline on when to start antiretroviral therapy and on pre-exposure prophylaxis for HIV. Geneva, Switzerland: WHO Press; 2015.
