# Supplementary material for: Determinants of first-line antiretroviral treatment failure among adult patients on treatment in Mettu Karl Specialized Hospital, South West Ethiopia; a case control study
Source: PLoS One. 2021 Oct 22;16(10):e0258930. doi: 10.1371/journal.pone.0258930 (PMC8535443; doi:10.1371/journal.pone.0258930)
Supplement: Attachment — Submitted filename: PONE-D-21-13362 commented.docx [file pone.0258930.s002.docx]

**Comments to the paper: PONE-D-21-13362**

**General**

The comments are described in the main manuscript with high-lighted texts and accessible to the author for possible amendments.

The paper is well articulated and relevant in addressing the current global health issue still imposing challenges to the globe even though showing promised changes.

**Specifically to mention some**,

**Title**

- Make it shorten if possible and to include hospital’s name

**Introduction**

- Abbreviated words/phrases should be explained fully at first appearance in the sentence, like ART, WHO, and the like.
- Distance of the study area from Addis Ababa, capital city of Ethiopia, not mentioned
- CD4 and viral load variables, how are they collected and analyzed, machine used never explained under data collection procedure

**Methodology**

- Study area is not fully described, distance/location from capital city not known.
- Sample size calculation not elaborated
- Ethical clearance and consent sub-section not included including approval board and number.
- Terms are not defined like adult, adherence (good, fair and poor), and the like

**Result**

- Table 1; others should be rechecked as it is greater than others; or else all mentioned variables should be listed out.
- Table2: p-value should be different from zero.
- Table 3: smoking and khat variables should be removed from that table. And also look for punctuation correction beneath the table

**Discussion**

- It becomes only one-sided discussion, only issue of similarity/consistency. Sometimes, disagreement is expected.
- Very recently published data/article (2021) not included in the study.

**Conclusion**

- It lacks recommendation

**Limitation**

- Not included as if the study is without limitation.

**References**

- Even though few, recent published data /online statistics for HIV-AIDS epidemiology not used/included.
