# Supplementary material for: Determinants of first-line antiretroviral treatment failure among adult patients on treatment in Mettu Karl Specialized Hospital, South West Ethiopia; a case control study
Source: PLoS One. 2021 Oct 22;16(10):e0258930. doi: 10.1371/journal.pone.0258930 (PMC8535443; doi:10.1371/journal.pone.0258930)
Supplement: Attachment — Submitted filename: Response to Reviewers.docx [file pone.0258930.s004.docx]

**Date 14/09/2021**

**To: PLOS ONE**

**Issue: Rebuttal Letter**

Dear all at the PLOS ONE and esteemed reviewers, we are very grateful to your academic inputs. We found all your comments and editorial recommendations very important for betterment of our paper. In regard to this, we prepared a rebuttal letter responding to all your comments separately for journal requirement and for the two reviewers. The specific editorial and review comments are fully accepted and changes are made in the manuscript accordingly. The responses are placed next to the provided comments for all in the next pages of this letter. We are ready to further refine our manuscript if you found some of your comments are insufficiently accommodated.

Regards

Sabit Zenu Siraj

Corresponding Author of the Manuscript

**Response to Journal Editorial Comments**

1. Please ensure that your manuscript meets PLOS ONE's style requirements, including those for file naming.

**Response**: The document is revised to fulfil PLOS ONE’s style and file naming requirements.

2. Please provide additional details regarding participant consent. In the ethics statement in the Methods and online submission information, please ensure that you have specified what type you obtained (for instance, written or verbal, and if verbal, how it was documented and witnessed). If your study included minors, state whether you obtained consent from parents or guardians. If the need for consent was waived by the ethics committee, please include this information.

**Response**: The required Ethics Statement is added to the manuscript file and to the online system. Manuscript, Page 9, Line 214-220.

3. We noted several instances in the manuscript where the P value equals zero; please correct and clarify.

**Response**: The P-Value in the table is corrected as P<0.01. Manuscript, Page 11, Table 3

4. We note that you have indicated that data from this study are available upon request. PLOS only allows data to be available upon request if there are legal or ethical restrictions on sharing data publicly.

**Response**: The data availability statement is changed and the data is currently supplemented as supplementary file.

5. Please include your full ethics statement in the ‘Methods’ section of your manuscript file. In your statement, please include the full name of the IRB or ethics committee who approved or waived your study, as well as whether or not you obtained informed written or verbal consent. If consent was waived for your study, please include this information in your statement as well.

**Response**: The required Ethics Statement is added to the manuscript file and to the online system. Manuscript, Page 9, Line 214-220.

6. During our internal evaluation of the manuscript, we found significant text overlap between your submission and the following previously published works, some of which you are an author.

**Response**: The observed similarity with the said online sources is accidental. The observed similarity is currently paraphrased. Manuscript, Page 4, Line 84-88

**Response to Reviewer 1**

**Reviewer Comments**

This is a well-written paper with a sound analysis, providing some interesting findings. The major contribution of this submission is that it is the first study in Ethiopia using viral load tests to confirm the correlates of non-adherence. Thus, while this study in Ethiopia identifies the barriers of adherence to first-line ART, there is not much new in the paper that has not been identified before. The method is sound and the study appears to have been nicely conducted. The results section is well-presented. I think this paper could be a publishable paper of some new insights within the Ethiopian context. However, I don't believe that PLOS one is the most appropriate venue as the journal includes pieces with broader focus and this paper may be a bit narrow. I would suggest that it should be published in a regional journal within Africa.

**Response:** Dear Reviewer, we appreciate your encouragement and it will help us in our future endeavours. Regarding the suitability of PLOS ONE for this particular work, we submitted it to this journal because the journal is one of the most reputable. It publishes high-quality papers that have undergone extensive editorial and peer reviews. In Ethiopia, senior researchers actively encourage novice researchers like me to publish their works on PLOS ONE. In addition, the journal published high volume of academic researchers from Africa. According to the current data, the journal published over 5000 researches of different types conducted in or by Africans. Considering this, we would be happy if this paper is published on PLOS ONE.

**Responses to Reviewer 2**

Dear Reviewer, we are very grateful to your academic inputs. We amended the document as per your comments and specific responses are listed hereunder.

**Title**

1. Make it shorten if possible and to include hospital’s name

**Response**: The title is shortened and the name of the hospital is included as ‘Determinants of first-line antiretroviral treatment failure among adult patients on treatment in Mettu Karl Specialized Hospital, South West Ethiopia; a case control study’. Manuscript, Title, Page: line 1-3

**Introduction:**

2. Abbreviated words/phrases should be explained fully at first appearance in the

**Response**: All abbreviation issues are corrected. Manuscript, Introduction, Page 3-5, Line 52-126

3. Distance of the study area from Addis Ababa, capital city of Ethiopia, not mentioned

**Response**: The distance and some detail is added to the study area in Materials and Methods section. Manuscript, Materials and Methods, Page 6, Line 128-140.

4. CD4 and viral load variables, how are they collected and analysed, machine used never explained under data collection procedure

**Response**: The materials used in the hospital are added to the Materials and Methods sections under data collection tools and procedures. Manuscript; Materials and Methods, Data collection tools and procedures, Page 8, Line 189-195.

**Methodology**

5. Study area is not fully described, distance/location from capital city not known.

**Response**: The distance and other detail are added to the study area in Materials and Methods section. Manuscript; Materials and Methods, Page 6, Line 128-140.

6. Sample size calculation not elaborated

**Response**: The sample size was calculated by using Epi Info 7 using an option for Unmatched Case Control study. Power 80%, 95% CI and other parameters were used to calculate the sample size. Further detail is added to the document. Manuscript; Materials and Methods (sample size determination and sampling technique) Page 6-7, Line 154-178.

7. Ethical clearance and consent sub-section not included including approval board and number.

**Response**: The recommended Ethics Statement is added to the manuscript file and to the online system. Manuscript; Methods and Materials, Ethics Statement, Page 9, Line 214-220.

8. Terms are not defined like adult, adherence (good, fair and poor), and the like

**Response**: The terms are operationalized as recommended. Manuscript; Materials and Methods, Operational Definitions, Page 8, Line 197-203.

**Result**

9. Table 1; others should be rechecked as it is greater than others; or else all mentioned variables should be listed out.

**Response**: Corrected in the document. Manuscript; Result, Table 2, Page 9.

10. Table2: p-value should be different from zero

**Response**: The P-Value in the table is corrected as P<0.01. Manuscript, Page 11, Table 3, the table is updated to table 3 as one more table is added to elaborate the sample size calculation procedures.

11. Table 3: smoking and khat variables should be removed from that table. And also look for punctuation correction beneath the table

**Response**: The variables are removed. The recommended changes are made to punctuations beneath the table. Manuscript; Result, Table 4, Page 13.

**Discussion**

12. It becomes only one-sided discussion, only issue of similarity/consistency. Sometimes, disagreement is expected.

**Response**: The recommended differences are added to the discussion. Manuscript; Discussion Page 26, line 358-369

13. Very recently published data/article (2021) not included in the study

**Response**: Recent UNAIDS 2021 report is added to the document and the information is updated accordingly.

**Conclusion**

14. It lacks recommendation

**Response**: Recommendation is added to the conclusion, Page 17, and Line 379-384

**Limitation**

15. Not included as if the study is without limitation.

**Response**: Limitation is added. Page 16, Line 370-372

**References**

16. Even though few, recent published data /online statistics for HIV-AIDS epidemiology not used/included.

**Response**: Updated epidemiological findings added to references. Page 18 and 19
